# Supplementary material for: Severe T cell hyporeactivity in ventilated COVID-19 patients correlates with prolonged virus persistence and poor outcomes
Source: Nat Commun. 2021 May 21;12:3006. doi: 10.1038/s41467-021-23334-2 (PMC8140132; doi:10.1038/s41467-021-23334-2)
Supplement: Supplementary file 1 — Supplementary Information [file 41467_2021_23334_MOESM1_ESM.pdf]

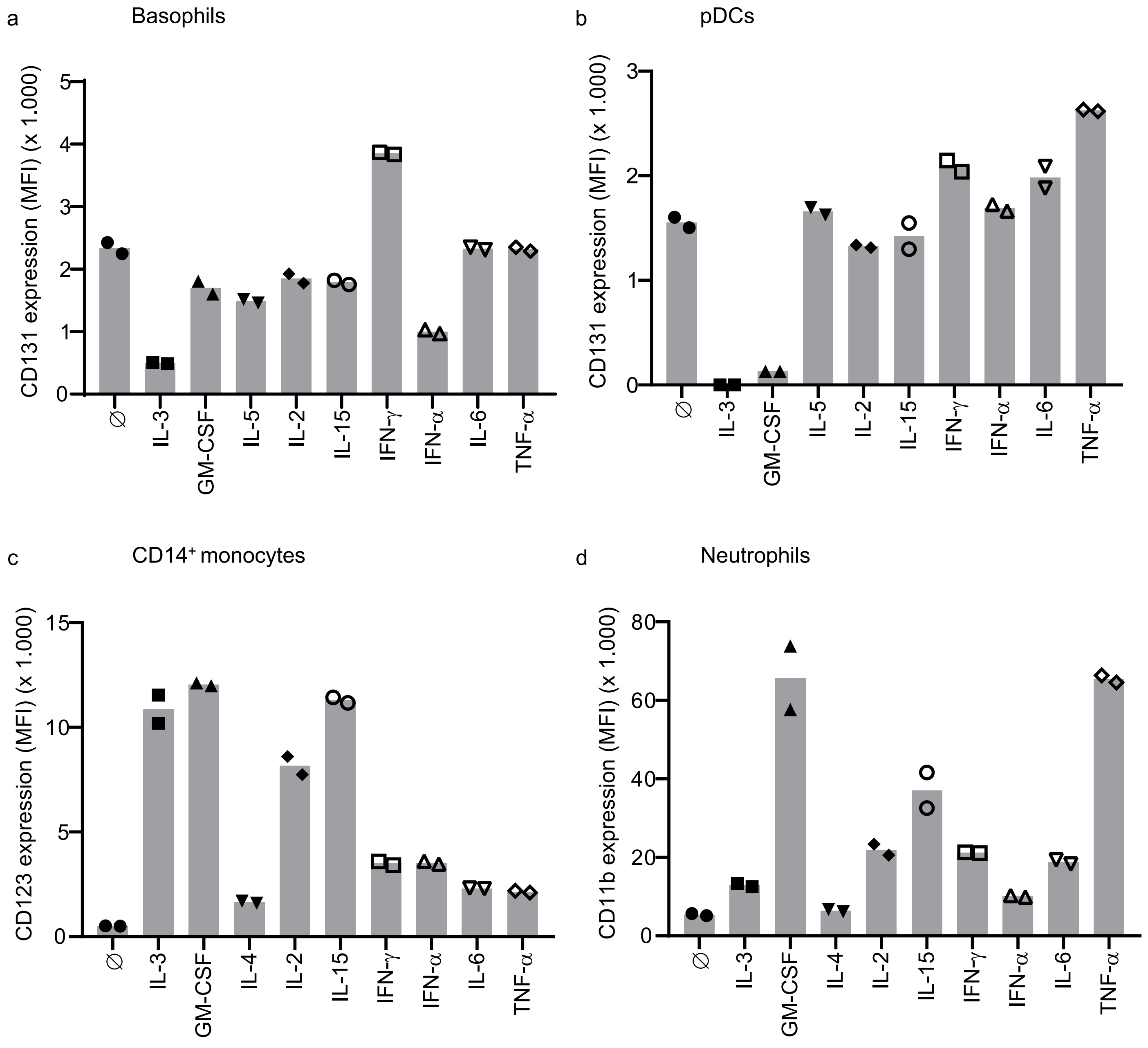
a
Basophils
CD131 expression (MFI) (x 1.000)
0
1
2
3
4
5
∅
IL-3
GM-CSF
IL-5
IL-2
IL-15
IFN-γ
IFN-α
IL-6
TNF-α
b
pDCs
CD131 expression (MFI) (x 1.000)
0
1
2
3
∅
IL-3
GM-CSF
IL-5
IL-2
IL-15
IFN-γ
IFN-α
IL-6
TNF-α
c
CD14+ monocytes
CD123 expression (MFI) (x 1.000)
0
5
10
15
∅
IL-3
GM-CSF
IL-4
IL-2
IL-15
IFN-γ
IFN-α
IL-6
TNF-α
d
Neutrophils
CD11b expression (MFI) (x 1.000)
0
20
40
60
80
∅
IL-3
GM-CSF
IL-4
IL-2
IL-15
IFN-γ
IFN-α
IL-6
TNF-α

Suppl. Fig. 1

Supplementary Fig. 1 Cytokine induced changes of surface markers in a whole blood assay. **a-d** Whole blood from a healthy donor was cultured in duplicates for 24 hours at 37°C with or without cytokines (IL-2, IL-3, IL-4, IL-5, IL-6, IL-15, GM-CSF, IFN-γ, IFN-α and TNF-α, 20 ng/ml each). Samples were analyzed by flow cytometry and absolute values of indicated cell surface markers are depicted as mean fluorescence intensity (MFI). **a** Expression of CD131 on basophils. **b** Expression of CD131 on pDCs. **c** Expression of CD123 on CD14+ monocytes. **d** Expression of CD11b on neutrophils. Bar graphs show mean values and each sample is represented by one dot. The experimental findings were successfully replicated twice. Source data are provided as a Source Data file.

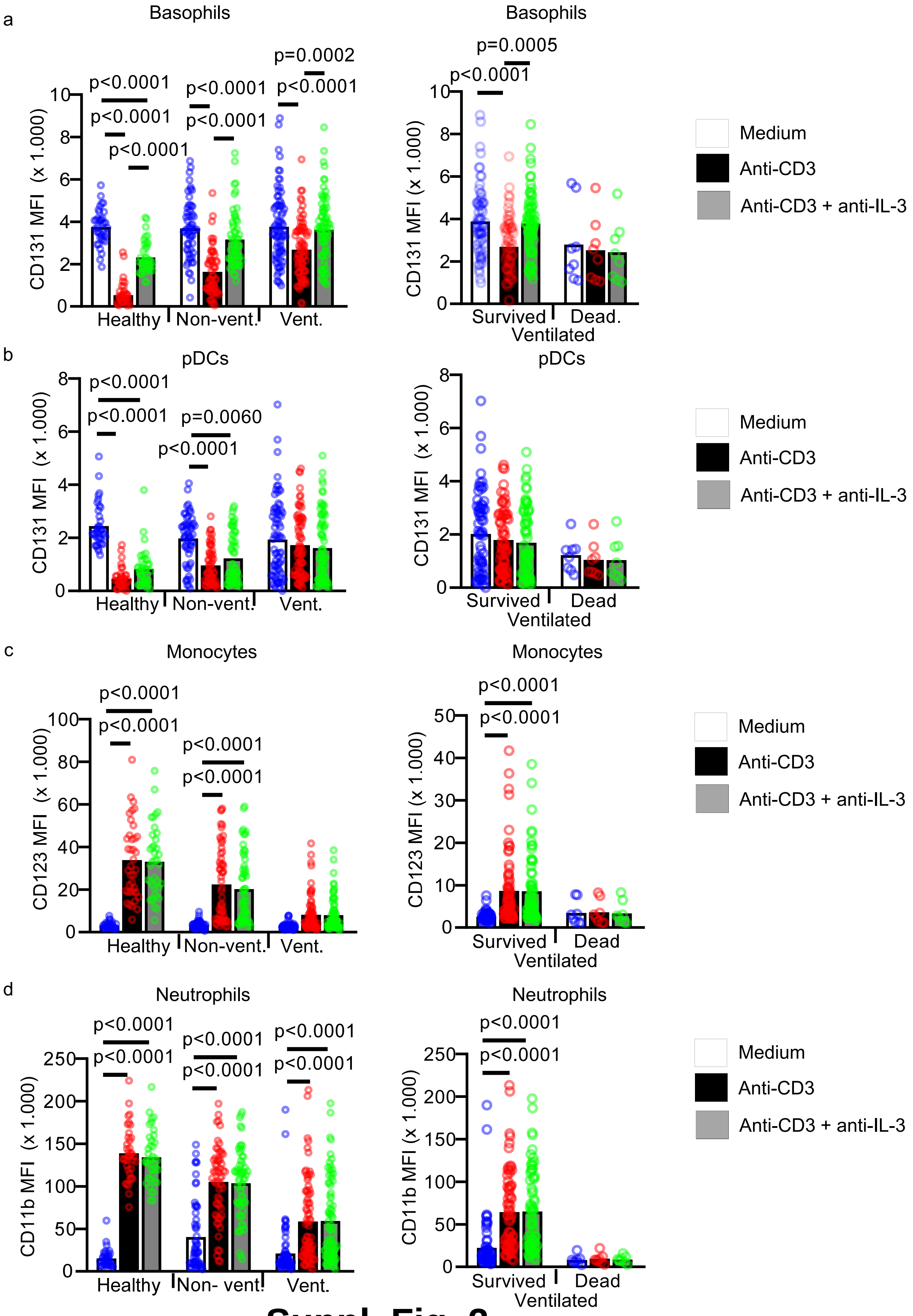
a
Basophils
CD131 MFI (x 1.000)
p<0.0001
p=0.0002
p=0.0005
Healthy
Non-vent.
Vent.
Survived
Dead.
Ventilated
Medium
Anti-CD3
Anti-CD3 + anti-IL-3
b
pDCs
p=0.0060
Dead
c
Monocytes
CD123 MFI (x 1.000)
d
Neutrophils
CD11b MFI (x 1.000)
Non- vent.

Suppl. Fig. 2

Supplementary Fig. 2 T cell reactivity in COVID-19 patients and healthy controls. a-d Whole blood from 38 healthy controls (Healthy; n=38 biologically independent samples), 33 non-ventilated (Non-vent.; n=58 biologically independent samples) and 21 mechanically ventilated (Vent.; n=77 biologically independent samples) COVID-19 patients was cultured without stimulation (medium), with immobilized anti-CD3, or with immobilized anti-CD3 plus anti-IL-3 (10 µg/ml) for 24h. Ventilated patients were stratified into "survived" (17 patients, n=69 biologically independent samples) and "dead" (4 patients, n=8 biologically independent samples). Expression of surface markers was quantified by flow cytometry and absolute expression values of indicated markers are shown as mean fluorescence intensity (MFI) on basophils (**a**), pDCs (**b**), CD14+ monocytes (**c**) and neutrophils (**d**). Bar graphs show mean values and each sample is represented by one dot. One-way ANOVA with Bonferroni multiple comparison test was used and statistical significance is only shown for differences between medium, anti-CD3 and anti-CD3+anti-IL-3 within each group of individuals. Source data are provided as a Source Data file.

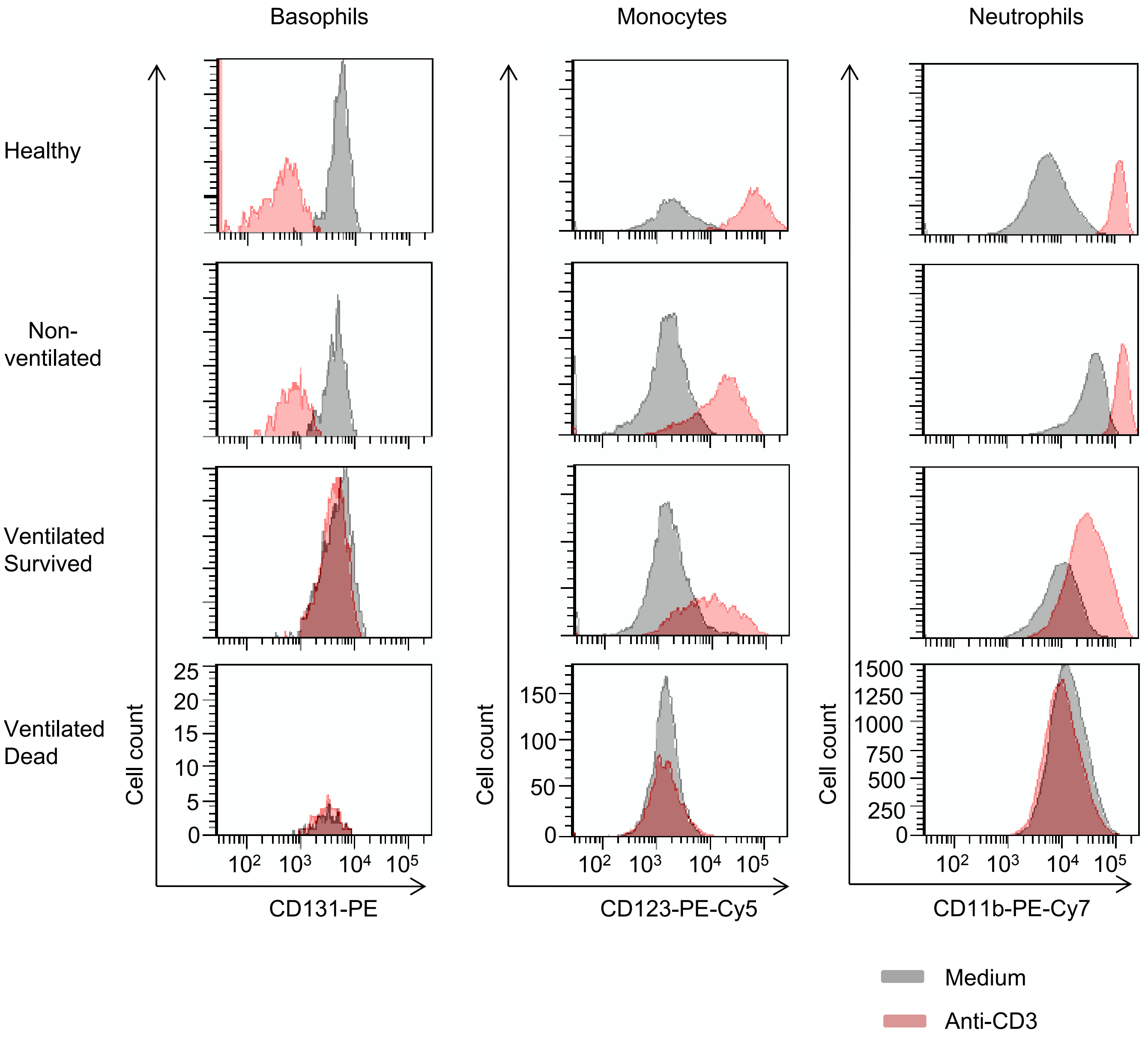

Basophils
Monocytes
Neutrophils
Healthy
Non-ventilated
Ventilated Survived
Ventilated Dead
Cell count
25
20
15
10
5
0
150
100
50
1500
1250
1000
750
500
250
10^2 10^3 10^4 10^5
CD131-PE
CD123-PE-Cy5
CD11b-PE-Cy7
Medium
Anti-CD3

Suppl. Fig. 3

Supplementary Fig. 3 Representative FACS histogram plots for whole blood stimulation. Whole blood from various donors as indicated was cultured with (red) or without (grey) immobilized anti-CD3 for 24h. Expression of surface markers was quantified by flow cytometry and representative histogram plots are shown.

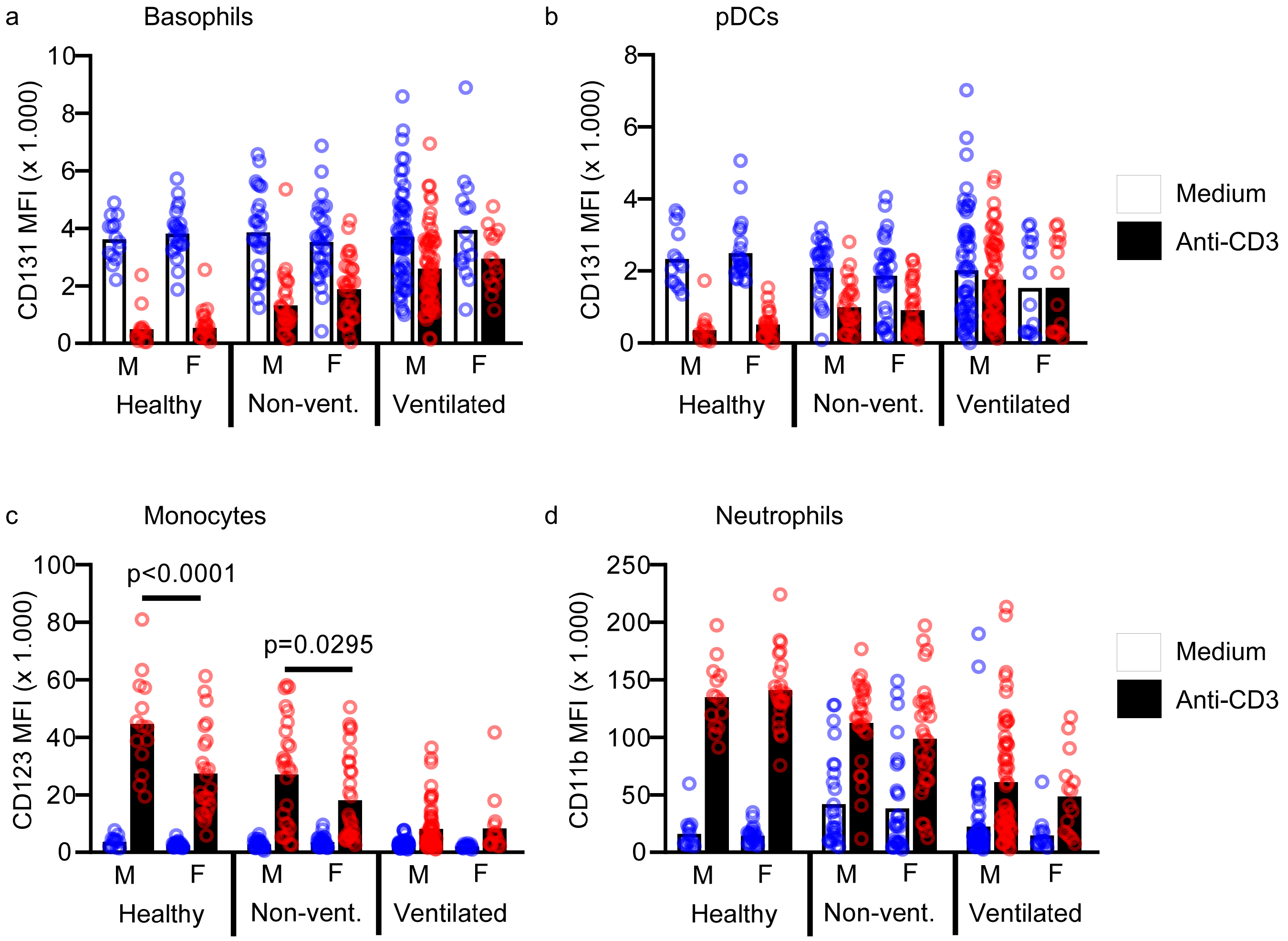

a
Basophils
CD131 MFI (x 1.000)
10
8
6
4
2
0
M
F
M
F
M
F
Healthy
Non-vent.
Ventilated
b
pDCs
CD131 MFI (x 1.000)
8
6
4
2
0
M
F
M
F
M
F
Healthy
Non-vent.
Ventilated
Medium
Anti-CD3
c
Monocytes
CD123 MFI (x 1.000)
100
80
60
40
20
0
p<0.0001
p=0.0295
M
F
M
F
M
F
Healthy
Non-vent.
Ventilated
d
Neutrophils
CD11b MFI (x 1.000)
250
200
150
100
50
0
M
F
M
F
M
F
Healthy
Non-vent.
Ventilated
Medium
Anti-CD3

Suppl. Fig. 4

Supplementary Fig. 4 Gender specific analysis of T cell activation and immunophenotypes. a-d Whole blood from healthy controls (n=14 biologically independent samples from 14 males and n=24 biologically independent samples from 24 females), non-ventilated COVID-19 patients (n=27 biologically independent samples from 16 males and n=31 biologically independent samples from 17 females) and mechanically ventilated COVID-19 patients (n=62 biologically independent samples from 16 males and n=15 biologically independent samples from 5 females) was cultured with or without immobilized anti-CD3 for 24h. Expression of surface markers was quantified by flow cytometry and absolute expression values of indicated markers are shown as mean fluorescence intensity (MFI) on basophils (**a**), pDCs (**b**), CD14+ monocytes (**c**) and neutrophils (**d**). Bar graphs show mean values and each sample is represented by one dot. One-way ANOVA with Bonferroni multiple comparison test was used and statistical significance is only shown for differences between males and females within each group of individuals. Source data are provided as a Source Data file.

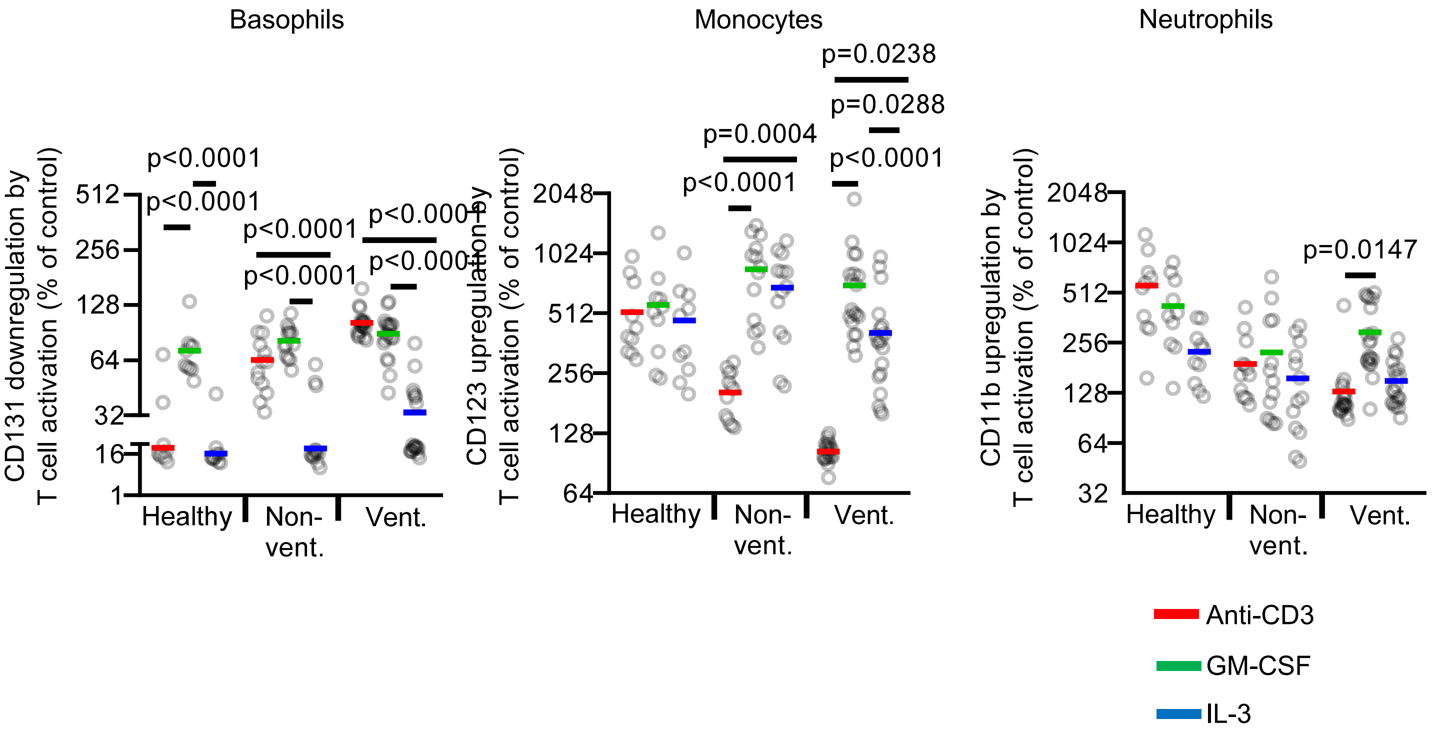
Basophils
Monocytes
Neutrophils
CD131 downregulation by T cell activation (% of control)
CD123 upregulation by T cell activation (% of control)
CD11b upregulation by T cell activation (% of control)
p<0.0001
p=0.0238
p=0.0288
p=0.0004
p=0.0147
Healthy
Non-vent.
Vent.
Anti-CD3
GM-CSF
IL-3

Suppl. Fig. 5

Supplementary Fig. 5 Effector cells of COVID-19 patients are not anergic to IL-3 or GM-CSF. Whole blood samples from 10 healthy controls (Healthy; n=10 biologically independent samples), 13 non-ventilated (Non-vent; n=13 biologically independent samples) and 15 mechanically ventilated (Vent.; n=19 biologically independent samples) COVID-19 patients were cultured with medium, anti-CD3, GM-CSF (10 ng/ml) or IL-3 (10 ng/ml) for 24h. Expression of indicated surface markers was quantified by flow cytometry on basophils, CD14+ monocytes and neutrophils. Values depict the ratio of surface marker expression with anti-CD3, GM-CSF or IL-3 to surface marker expression with medium alone. Each sample is represented by one dot and the mean is marked in red (anti-CD3), green (GM-CSF) or blue (IL-3). One-way ANOVA with Bonferroni multiple comparison test was used. Source data are provided as a Source Data file.

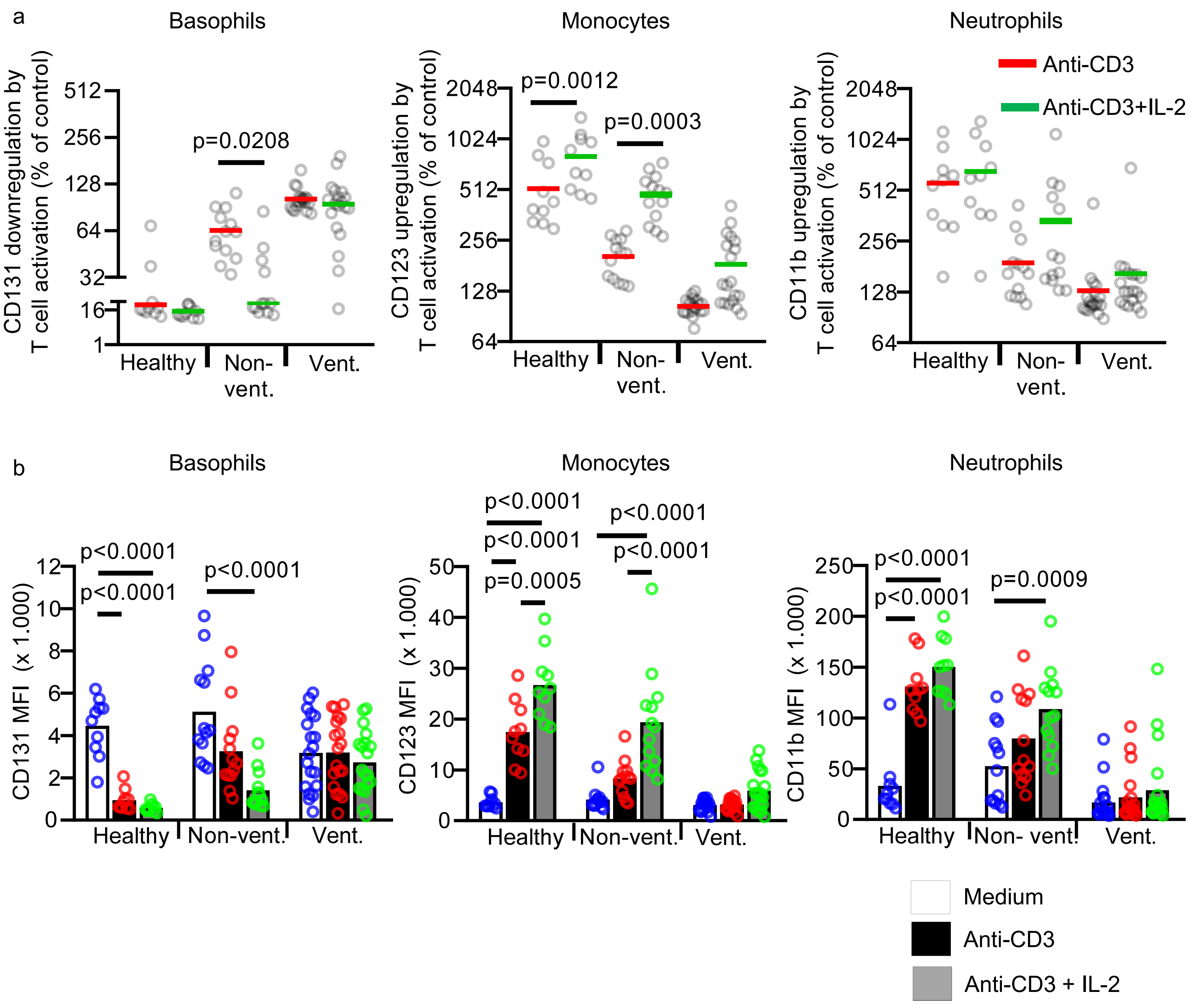

a
Basophils
Monocytes
Neutrophils
CD131 downregulation by T cell activation (% of control)
512
256
128
64
32
16
1
p=0.0208
Healthy
Non-vent.
Vent.
CD123 upregulation by T cell activation (% of control)
2048
1024
512
256
128
64
p=0.0012
p=0.0003
CD11b upregulation by T cell activation (% of control)
Anti-CD3
Anti-CD3+IL-2
b
Basophils
Monocytes
Neutrophils
CD131 MFI (x 1.000)
p<0.0001
p<0.0001
p<0.0001
CD123 MFI (x 1.000)
p<0.0001
p<0.0001
p=0.0005
p<0.0001
p<0.0001
CD11b MFI (x 1.000)
p<0.0001
p<0.0001
p=0.0009
Healthy
Non-vent.
Vent.
Non- vent.
Medium
Anti-CD3
Anti-CD3 + IL-2

Suppl. Fig. 6

Supplementary Fig. 6 Effect of IL-2 on T cell reactivity in COVID-19 patients and healthy controls. a-b Whole blood samples from 10 healthy controls (Healthy; n=10 biologically independent samples), 13 non-ventilated (Non-vent; n=13 biologically independent samples) and 15 mechanically ventilated (Vent.; n=19 biologically independent samples) COVID-19 patients were cultured with medium alone, with anti-CD3 or with anti-CD3 plus IL-2 (20 ng/ml) for 24h. Expression of indicated surface markers was quantified by flow cytometry on basophils, CD14+ monocytes and neutrophils (**a**). Values depict the ratio of surface marker expression with anti-CD3 or anti-CD3 plus IL-2 to surface marker expression without anti-CD3. Each sample is represented by one dot and the mean is marked in red (anti-CD3) or green (anti-CD3+IL-2). The absolute expression values of indicated markers are shown as mean fluorescence intensity (MFI) (**b**). Bar graphs show mean values and each sample is represented by one dot. One-way ANOVA with Bonferroni multiple comparison test was used. Source data are provided as a Source Data file.

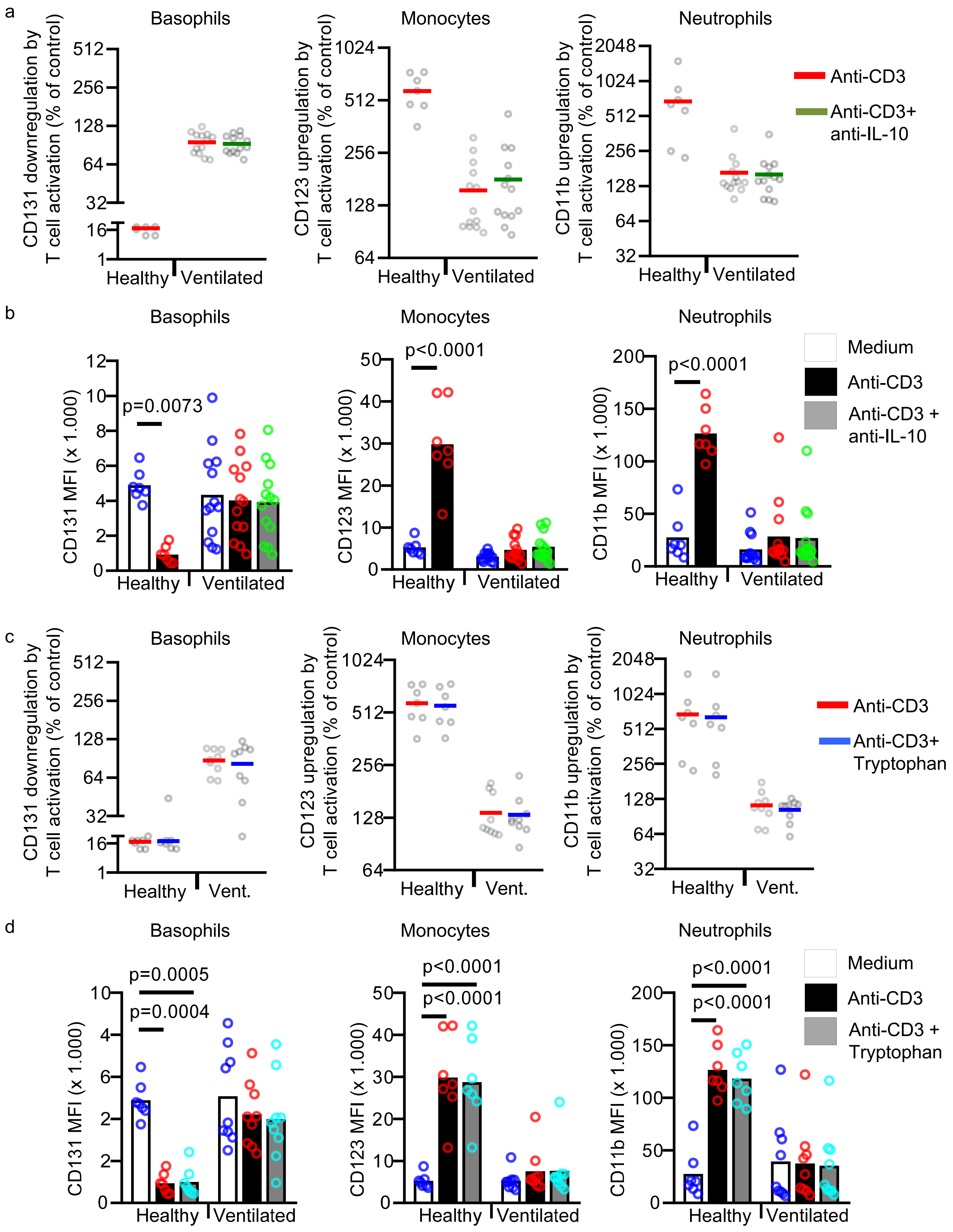
a
Basophils
Monocytes
Neutrophils
CD131 downregulation by T cell activation (% of control)
CD123 upregulation by T cell activation (% of control)
CD11b upregulation by T cell activation (% of control)
Healthy
Ventilated
Anti-CD3
Anti-CD3+ anti-IL-10
b
CD131 MFI (x 1.000)
CD123 MFI (x 1.000)
CD11b MFI (x 1.000)
p=0.0073
p<0.0001
p<0.0001
Medium
Anti-CD3
Anti-CD3 + anti-IL-10
c
Vent.
Anti-CD3
Anti-CD3+ Tryptophan
d
p=0.0005
p=0.0004
p<0.0001
p<0.0001
p<0.0001
p<0.0001
Medium
Anti-CD3
Anti-CD3 + Tryptophan

Suppl. Fig. 7

Supplementary Fig. 7 IL-10 and tryptophan degradation do not explain T cell hyporeactivity in COVID-19 patients. a-b Whole blood samples from 7 healthy controls (Healthy; n=7 biologically independent samples) and 13 mechanically ventilated (Vent.; n=13 biologically independent samples) COVID-19 patients were cultured with medium alone, with anti-CD3 or with anti-CD3 plus a blocking anti-IL-10 antibody (10 µg/ml) for 24h. Expression of indicated surface markers was quantified by flow cytometry on basophils, CD14+ monocytes and neutrophils. (**a**) Values depict the ratio of surface marker expression with anti-CD3 or anti-CD3 plus anti-IL-10 to surface marker expression without anti-CD3. Each sample is represented by one dot and the mean is marked in red (anti-CD3) or green (anti-CD3+anti-IL-10). **(b)** The absolute expression values of indicated markers are shown as mean fluorescence intensity (MFI). Bar graphs show mean values and each samples is represented by one dot. **c-d** Whole blood samples from 7 healthy controls (Healthy; n=7 biologically independent samples) and 9 mechanically ventilated (Vent.; n=9 biologically independent samples) COVID-19 patients were cultured with medium alone, with anti-CD3 or with anti-CD3 plus L-tryptophan (100 µg/ml) for 24h. Expression of indicated surface markers was quantified by flow cytometry on basophils, CD14+ monocytes and neutrophils. (**c**) Values depict the ratio of surface marker expression with anti-CD3 or anti-CD3 plus L-tryptophan to surface marker expression without anti-CD3. Each sample is represented by one dot and the mean is marked in red (anti-CD3) or blue (anti-CD3+L-tryptophan). The absolute expression values of indicated markers are shown as mean fluorescence intensity (MFI) (**d**). Bar graphs show mean and each sample is represented by one dot. One-way ANOVA with Bonferroni multiple comparison test was used. Source data are provided as a Source Data file.

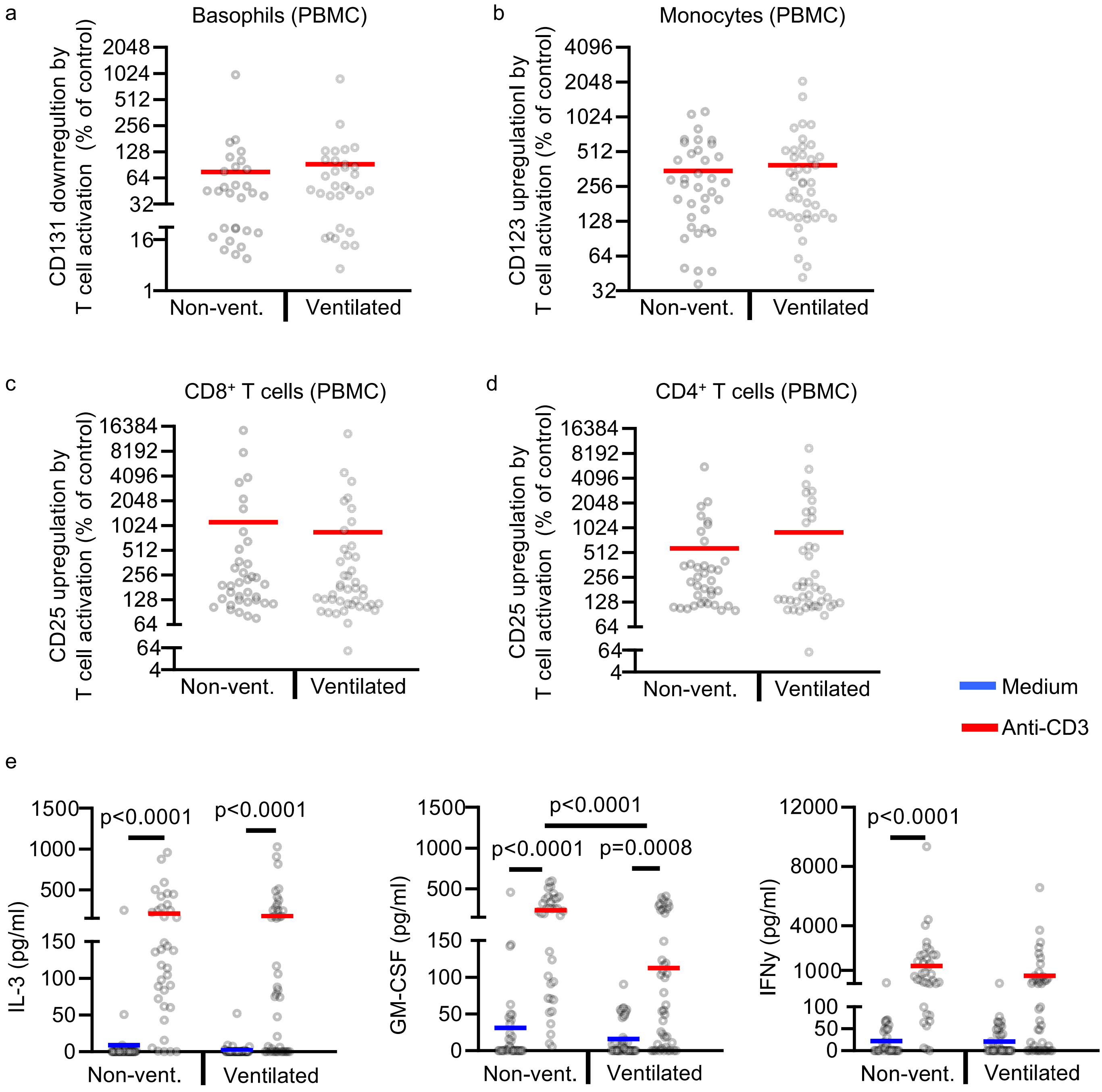
a
Basophils (PBMC)
CD131 downregultion by T cell activation (% of control)
2048
1024
512
256
128
64
32
16
1
Non-vent.
Ventilated
b
Monocytes (PBMC)
CD123 upregulationl by T cell activation (% of control)
4096
2048
1024
512
256
128
64
32
Non-vent.
Ventilated
c
CD8+ T cells (PBMC)
CD25 upregulation by T cell activation (% of control)
16384
8192
4096
2048
1024
512
256
128
64
64
4
Non-vent.
Ventilated
d
CD4+ T cells (PBMC)
CD25 upregulation by T cell activation (% of control)
16384
8192
4096
2048
1024
512
256
128
64
64
4
Non-vent.
Ventilated
Medium
Anti-CD3
e
IL-3 (pg/ml)
1500
1000
500
150
100
50
0
p<0.0001
p<0.0001
Non-vent.
Ventilated
GM-CSF (pg/ml)
1500
1000
500
150
100
50
0
p<0.0001
p<0.0001
p=0.0008
Non-vent.
Ventilated
IFNy (pg/ml)
12000
8000
4000
1000
100
50
0
p<0.0001
Non-vent.
Ventilated

Suppl. Fig. 8

Supplementary Fig. 8 T cell reactivity analyzed with PBMC from COVID-19 patients. a-e PBMCs from 25 non-ventilated COVID-19 patients (Non-vent.; n=36 biologically independent samples) and 16 mechanically ventilated COVID-19 patients (Ventilated, n=42 biologically independent samples) were cultured with or without anti-CD3 (5 µg/ml) for 24h. Analysis of basophils was not possible in all samples because basophil numbers were too low in some samples. Expression of indicated surface markers was quantified by flow cytometry on basophils (**a**), CD14+ monocytes (**b**), CD8+ T cells (**c**) and CD4+ T cells (**d**). Values depict the ratio of surface marker expression with anti-CD3 to surface marker expression without anti-CD3 in percent. Each sample is represented by one dot and the mean is marked in red. **e** Concentrations of IL-3, GM-CSF and IFN-γ were measured in the culture supernatant by ELISA. Each sample is represented by one dot and the mean is marked in blue (Medium) or red (anti-CD3). 2-tailed unpaired t-test (a-d) or one-way ANOVA with Bonferroni multiple comparison test (e) was used. Source data are provided as a Source Data file.

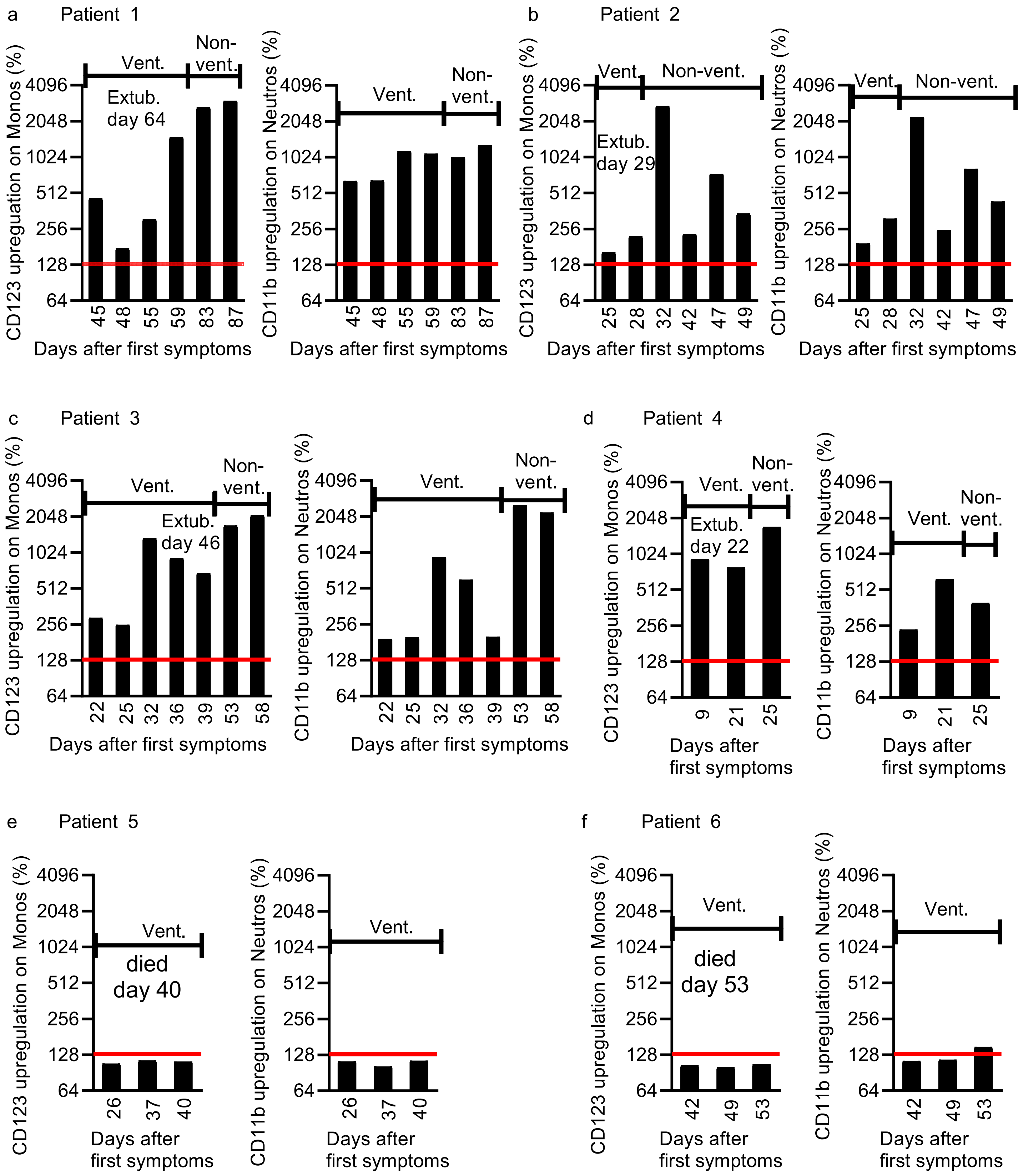
a Patient 1
b Patient 2
c Patient 3
d Patient 4
e Patient 5
f Patient 6
CD123 upregulation on Monos (%)
CD11b upregulation on Neutros (%)
Days after first symptoms
Vent.
Non-vent.
Extub. day 64
Extub. day 29
Extub. day 46
Extub. day 22
died day 40
died day 53

Suppl. Fig. 9

Supplementary Fig. 9 Examples of longitudinal analysis of ventilated COVID-19 patients. Upregulation of CD123 on CD14+ monocytes and CD11b on neutrophils in whole blood cultures with immobilized anti-CD3. Values depict the ratio of surface marker expression with anti-CD3 to surface marker expression without anti-CD3. 4 patients could be weaned from mechanical ventilation (patient 1-4) and 2 patients died on the ICU (patient 5-6). Periods of ventilation and non-ventilation are marked as “Vent.” and “Non-vent”. The cut-off of 130% upregulation that was used for the predictive score is shown as red line. Source data are provided as a Source Data file.

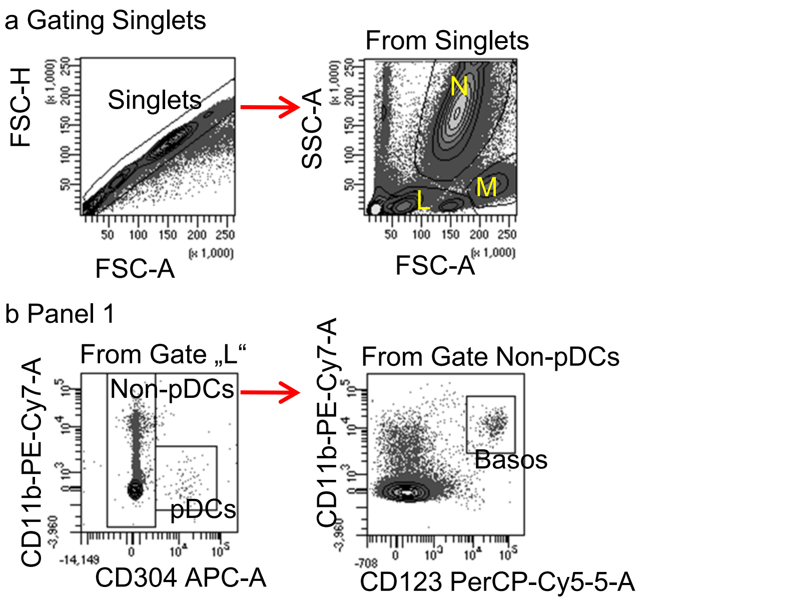

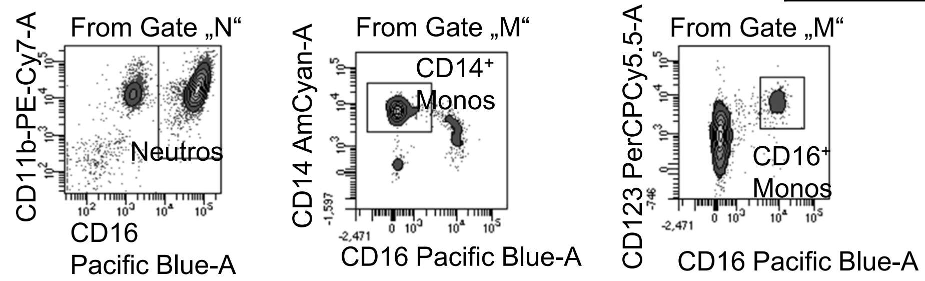

Panel 1:

Antibody	Format
CD116	FITC
CD123	PE-Cy5
CD131	PE
CD304	APC
CD11b	PE-Cy7
CD193	APC-Cy7
CD16	Pacific Blue
CD14	V500

Panel 2:

Antibody	Format
CD116	FITC
CD123	PE-Cy5
CD131	PE
HLA-DRII	APC
CD8	PE-Cy7
CD3	APC-Cy7
CD19	Pacific Blue
CD4	V500

Panel 3:

Antibody	Format
CD116	FITC
CD169	PE
CD3	APC-Cy7
CD16	Pacific Blue
CD14	V500

Panel 4:

Antibody	Format
HLA-DRII	FITC
CD123	PE-Cy5
CD131	PE
CD25	APC
CD11b	PE-Cy7
CD8	APC-Cy7
CD16	Pacific Blue
CD4	V500

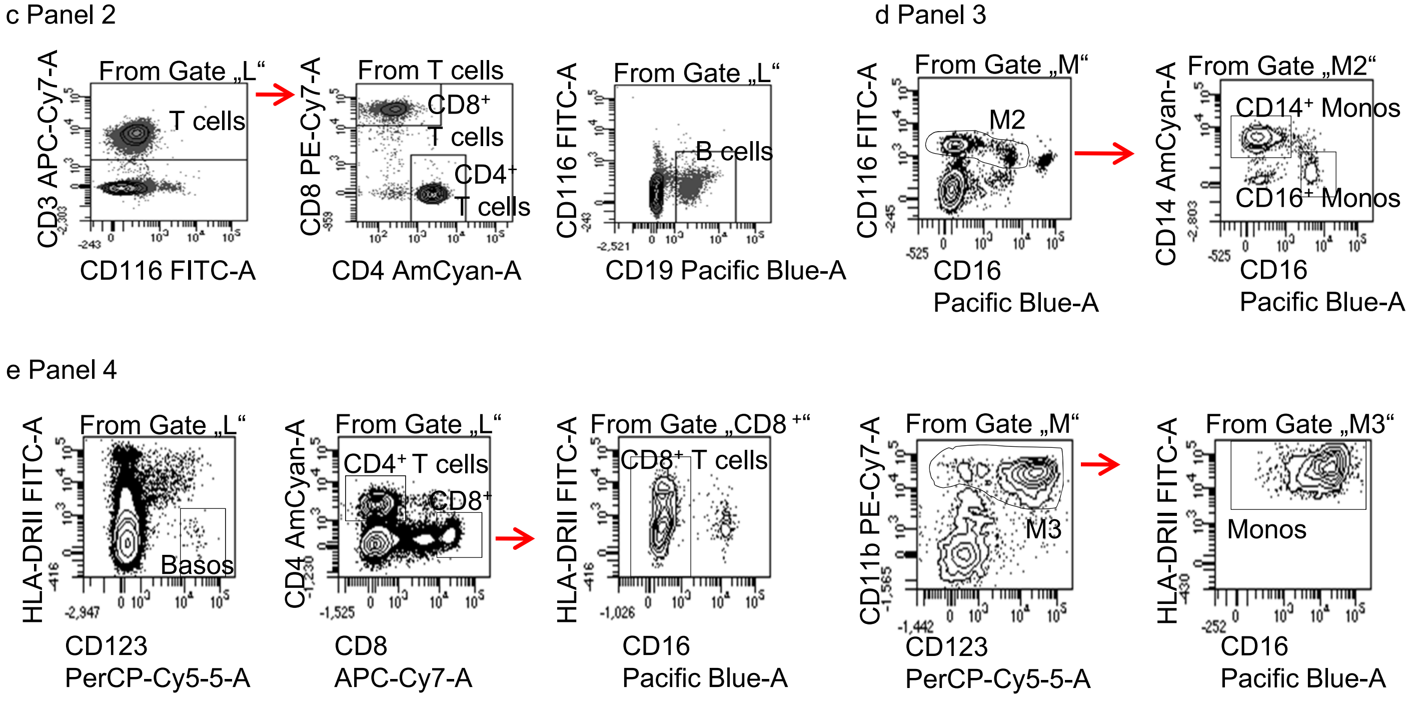

Suppl. Fig. 10

Supplementary Fig. 10 Gating strategy to detect key leukocyte subpopulations by flow cytometry. (**a**) Gating strategy of single cells. (**b**) Panel 1: Gating strategy to identify pDCs, basophils, neutrophils and CD14+ monocytes, CD16+ monocytes used for Fig. 1a-f, Fig. 3, Fig. Fig. 4 a-d, Suppl. Fig.1a-d, Suppl. Fig. 2a-d, Suppl. Fig. 3, Suppl. Fig. 4a-d, Suppl. Fig. 5, Suppl. Fig. 6a-b, Suppl. Fig. 7a-d, Suppl. Fig. 9. (**c**) Panel 2: Gating strategy to identify CD4+ and CD8+ T cells used for Fig. 2a-b. (**d**) Panel 3: Gating strategy to identify CD14+ monocytes and CD16+ monocytes used for Fig. 4e-h. (**e**) Panel 4: Gating strategy to identify basophils, CD4+ T cells and CD8+ T cells and monocytes from cultured PBMCs (Suppl. Fig. 8a-d).

	Healthy	Covid-19 all	Covid-19 non-ventilated	Covid-19 ventilated all	Covid-19 ventilated survived	Covid-19 ventilated dead
**Laboratory values: (demographics)**						
Number of samples (n)	42	188	68	120	101	19
Number of patients (n)	42	55	39 #	30	23	7
**Laboratory values: (mean)**						
Procalcitonin (ng/ml)		1.6	1.1	1.7	1.1	4.5 [1]
CRP (mg/l)		69.6	35.6	86.1 [2]	76.1	141,2 [3]
IL-6 (pg/ml)		85.0	23.9	94.3	71.1	216,4 [4]
Ferritin (ng/ml)		2721.9	1158.2	3018.5	1561.6	10950,4 [5]
LDH		340.2	273.9	370.6 [6]	361.1	423,3
ALAT (U/L)		75.0	62.9	80.7	74.0	116,6 [7]
Bilirubin (mg/dl)		2.2	0.9	2.8 [8]	0.9	12,5 [9]
CK (U/L)		125.7	65.5	153.1	156.7	132,6
D-Dimer (mg/L)		8.2	4.0	8.6 [10]	9.0	6,4
Leucocytes (/nl)		13.0	11,4	13.8	11.5	25,8 [11]

14 patients were sampled on ventilation and also after weaning.
[1] p=0.0011; [2] p=0.0001; [3] p=0.0052; [4] p= 0.0008; [5] p< 0.0001; [6] p= 0.0002; [7] p=0.0210; [8] p= 0.0420; [9] p< 0.0001; [10] p= 0,0317; [11] p= 0.0006

Suppl. Tab. 1

Supplementary Tab. 1 Clinical laboratory characteristics of COVID-19 patients and healthy controls. COVID-19 patients were sub-grouped into non-ventilated and ventilated patients. Ventilated patients were further sub-grouped into "Survived" and "Dead". In most patients several consecutive blood samples were available. 14 patients were first sampled on ventilation and also after weaning from ventilation. 2-tailed unpaired t-test was used to calculate statistical differences between non-ventilated and ventilated patients as well as between "Survived" and "Dead" patients. 2-tailed unpaired t-test was used to calculate statistical differences.

	Virus persistence in days (mean +/- SEM)	Duration of T cell hyporeactivity in days (mean +/- SEM), measured by CD123 upregulation on monocytes	Duration of T cell hyporeactivity in days (mean +/- SEM), measured by CD11b upregulation on neutrophils
Patients with virus persistence < 15 days	5.8 (± 1.1)	5.3 (± 2.7)	10.6 (± 3.3)
Patients with virus persistence ≥ 15 days	31.0 *** (± 3.1)	24.0 ** (± 4.8)	23.9 * (± 4.6)

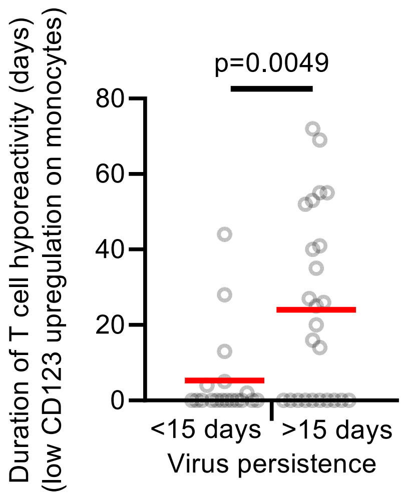

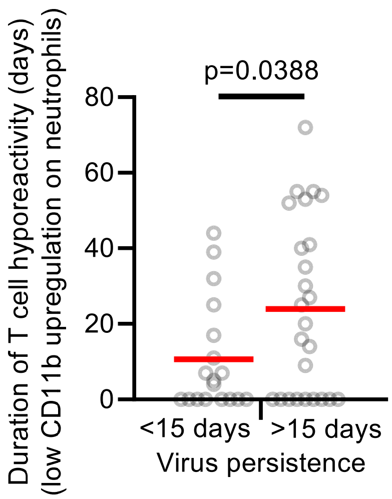

Suppl. Tab. 2

Supplementary Tab. 2 Correlation between persistence of SARS-CoV-2 replication and duration of T cell hyporeactivity. Virus persistence was defined as the period from the day of first clinical symptoms to the last day of positive virus RT-PCR. Patients were stratified in two groups by virus persistence <15 days (n=18 biologically independent samples) or ≥15 days (n=25 biologically independent samples). T cell reactivity was quantified by upregulation of CD123 on monocytes or CD11b on neutrophils as described in Fig. 1. T cell hyporeactivity was defined as an upregulation < 300% for both surface markers. Duration of T cell hyporeactivity was defined as the period from the day of first clinical symptoms to the last day of T cell hyporeactivity.

The figure below shows individual data points for duration of T cell hyporeactivity and virus persistence <15 days or ≥15 days. 2-tailed unpaired t-test was used to calculate statistical differences between the two groups of patients. Source data are provided as a Source Data file.
