# Supplementary material for: Severe T cell hyporeactivity in ventilated COVID-19 patients correlates with prolonged virus persistence and poor outcomes
Source: Nat Commun. 2021 May 21;12:3006. doi: 10.1038/s41467-021-23334-2 (PMC8140132; doi:10.1038/s41467-021-23334-2)
Supplement: Supplementary file 2 — Reporting summary [file 41467_2021_23334_MOESM2_ESM.pdf]

nature research

Corresponding author(s): Matthias Mack

Last updated by author(s): Apr 6, 2021

Reporting Summary

Nature Research wishes to improve the reproducibility of the work that we publish. This form provides structure for consistency and transparency in reporting. For further information on Nature Research policies, see our Editorial Policies and the Editorial Policy Checklist.

Statistics

For all statistical analyses, confirm that the following items are present in the figure legend, table legend, main text, or Methods section.

n/a	Confirmed	
☐	☒	The exact sample size (n) for each experimental group/condition, given as a discrete number and unit of measurement
☐	☒	A statement on whether measurements were taken from distinct samples or whether the same sample was measured repeatedly
☐	☒	The statistical test(s) used AND whether they are one- or two-sided<br>*Only common tests should be described solely by name; describe more complex techniques in the Methods section.*
☒	☐	A description of all covariates tested
☐	☒	A description of any assumptions or corrections, such as tests of normality and adjustment for multiple comparisons
☐	☒	A full description of the statistical parameters including central tendency (e.g. means) or other basic estimates (e.g. regression coefficient) AND variation (e.g. standard deviation) or associated estimates of uncertainty (e.g. confidence intervals)
☐	☒	For null hypothesis testing, the test statistic (e.g. F, t, r) with confidence intervals, effect sizes, degrees of freedom and P value noted<br>*Give P values as exact values whenever suitable.*
☒	☐	For Bayesian analysis, information on the choice of priors and Markov chain Monte Carlo settings
☒	☐	For hierarchical and complex designs, identification of the appropriate level for tests and full reporting of outcomes
☒	☐	Estimates of effect sizes (e.g. Cohen's d, Pearson's r), indicating how they were calculated

Our web collection on statistics for biologists contains articles on many of the points above.

Software and code

Policy information about availability of computer code

Data collection	FACS DIVA 8 Software BD Biosciences
Data analysis	GraphPad Prism 8 Software

For manuscripts utilizing custom algorithms or software that are central to the research but not yet described in published literature, software must be made available to editors and reviewers. We strongly encourage code deposition in a community repository (e.g. GitHub). See the Nature Research guidelines for submitting code & software for further information.

Data

Policy information about availability of data

All manuscripts must include a data availability statement. This statement should provide the following information, where applicable:

- Accession codes, unique identifiers, or web links for publicly available datasets
- A list of figures that have associated raw data
- A description of any restrictions on data availability

The datasets generated during and/or analyzed during the current study are available from the corresponding author on reasonable request.

Field-specific reporting

Life sciences study design

All studies must disclose on these points even when the disclosure is negative.

Sample size	No statistical methods were used to calculate the sample size. Sample size was determined based on the number of patients admitted to the University Hopsital of Regensburg between March to July 2020 that consented. Clinical specimens were collected approximately every 4 days where an individuals´s clinical status permitted, and was continued until patient discharge or death.
Data exclusions	If data were excluded, this is described in the figure legends or source data file. Data were only excluded, when the number of pDCs was too low or not present at all to be analyzed (Suppl. Fig. 4b and Suppl. Fig 2b) or samples were spilled (2 samples in Suppl. Fig. 8e)
Replication	Supplementary figure 1: experimental findings were successfully replicated (2x). In all other cases no replications were done, because only one sample per patient per day was available.
Randomization	No randomisation was performed for the human data as this was not an interventional clinical study. Samples of patients were included in the study, if the inclusion criteria (e.g. SARS-CoV-2 RNA detection by qPCR) were fulfilled.
Blinding	The investigators that performed the experimental assays were blinded, as the samples were made available via a central facility and unblinding of sample codes was done later.

Reporting for specific materials, systems and methods

We require information from authors about some types of materials, experimental systems and methods used in many studies. Here, indicate whether each material, system or method listed is relevant to your study. If you are not sure if a list item applies to your research, read the appropriate section before selecting a response.

Materials & experimental systems

n/a	Involved in the study	
☐	☒	Antibodies
☒	☐	Eukaryotic cell lines
☒	☐	Palaeontology and archaeology
☒	☐	Animals and other organisms
☐	☒	Human research participants
☒	☐	Clinical data
☒	☐	Dual use research of concern

Methods

n/a	Involved in the study	
☒	☐	ChIP-seq
☐	☒	Flow cytometry
☒	☐	MRI-based neuroimaging

Antibodies

Antibodies used	All antibodies used in this study are against human proteins: anti-hCD3 APC-Cy7 (clone: SK7, BioLegend, Cat.# 344818, dilution 1:100), anti-hCD4 V500 (clone RPA-T4, BD Bioscience, Cat.# 560768, dilution 1:100), anti-hCD8 APC-eFluor 780 (clone: RPA-T8, Invitrogen, Darmstadt, Germany, Cat.# 47-0088-42, dilution 1:40), anti-hCD8 PE-Cy7 (clone: SK1, BioLegend, Cat.# 344712, dilution 1:100), anti-hCD11b PE-Cy7(clone: M1/70, BioLegend, San Diego, CA, Cat.# 101216, dilution 1:100), anti-hCD14 V500 (clone: MΦP9, BD Bioscience, Cat.# 562693, dilution 1:100), anti-hCD16 Pacific Blue (clone: 3G8, BioLegend, Cat.# 302032, dilution 1:100), anti-hCD19 Pacific Blue (clone: HIB19, BioLegend, Cat.# 302232, dilution 1:100), anti-hCD25 APC (clone: BC96, BioLegend, Cat.# 302610, dilution 1:40), anti-hCD116 FITC (clone: 4H1, BioLegend, Cat.# 305906, dilution 1:40), anti-hCD131 PE (clone: 1C1, eBioscience, Cat.# 12-1319-42, dilution 1:100), anti-hCD123 PE-Cy5 (clone: 9F5, BD Bioscience, Cat.# 551065, dilution 1:20), anti-hCD169 PE (clone: 7-239, Miltenyi Biotec, Bergisch Gladbach, Germany, Cat.# 130-098-654, dilution 1:22), anti-hCD304 APC (clone: 12C2, BioLegend, Cat.# 354506, dilution 1:40), anti-hHLA-DR II APC (clone: G-46-6, BD Bioscience, Cat.# 559866, dilution 1:100) and FITC (clone: G-46-6, BD Bioscience, Cat.# 555811, dilution 1:100). Anti-CD3 (clone OKT3, eBiosciences, San Diego, CA, Catalog # 16-0037-85) was used at 5 µg/ml. The blocking anti-IL-3 antibody (clone P8C11) was generated in our lab and used at a concentration of 10 µg/ml. The blocking anti-IL-10 antibody (clone JES3-19F1, BioLegend, San Diego, USA, Cat.# 506813) was used at 20 µg/ml.
Validation	FACS: All antibodies used in this study are commercially available, and all have been validated by the manufacturers and used by other publications. Likewise, we titrated these antibodies according to our own staining conditions. anti-hCD3 APC-Cy7 (clone: SK7, BioLegend, Cat.# 344818) (Human, Chimpanzee), anti-hCD4 V500 (clone RPA-T4, BD Bioscience, Cat.# 560768) (Human), anti-hCD8 APC-eFluor 780 (clone: RPA-T8, Invitrogen, Darmstadt, Germany, Cat.# 47-0088-42) (Human, Rat), anti-hCD8 PE-Cy7 (clone: SK1, BioLegend, Cat.# 344712) (Human, African Green, Chimpanzee, Cynomolgus, Pigtailed Macaque, Rhesus, Sooty Mangabey), anti-hCD11b PE-Cy7(clone: M1/70, BioLegend, San Diego, CA, Cat.# 101216) (Mouse, Human, Chimpanzee, Baboon, Cynomolgus, Rhesus, Rabbit (Lapine)), anti-hCD14 V500 (clone: MΦP9, BD Bioscience, Cat.# 562693) (Human), anti-hCD16 Pacific Blue (clone: 3G8, BioLegend, Cat.# 302032) (Human, African Green, Baboon, Capuchin Monkey, Chimpanzee, Cynomolgus, Marmoset, Pigtailed Macaque, Rhesus, Sooty Mangabey, Squirrel Monkey), anti-hCD19 Pacific Blue (clone: HIB19, BioLegend, Cat.# 302232) (Human, Chimpanzee, Rhesus), anti-hCD25 APC (clone: BC96, BioLegend, Cat.# 302610) (Human, Baboon, Chimpanzee, Pigtailed Macaque, Rhesus), anti-hCD116 FITC (clone: 4H1, BioLegend, Cat.# 305906) (Human, African Green), anti-hCD131 PE (clone: 1C1, eBioscience, Cat.# 12-1319-42) (Human), anti-hCD123 PE-Cy5 (clone: 9F5, BD Bioscience, Cat.# 551065) (Human), anti-hCD169 PE (clone: 7-239, Miltenyi Biotec, Bergisch Gladbach, Germany, Cat.# 130-098-654) (Human), anti-hCD304 APC (clone: 12C2, BioLegend, Cat.# 354506) (Human), anti-hHLA-DR II APC (clone: G-46-6, BD Bioscience, Cat.# 559866) (Human, Rhesus, Cynomolgus, Baboon, Dog) and FITC (clone: G-46-6, BD Bioscience, Cat.# 555811) (Human, Rhesus, Cynomolgus, Baboon, Dog). Anti-CD3 (clone OKT3, eBiosciences, San

Diego, CA, Catalog # 16-0037-85) (Human, Mouse) was used at 5 µg/ml. The blocking anti-IL-10 antibody (clone JES3-19F1, BioLegend, San Diego, USA, Cat.# 506813) (Human) was used at 20 µg/ml

Human research participants

Policy information about studies involving human research participants

Population characteristics	55 adult patients diagnosed with COVID-19 and 42 adult healthy volunteers without any clinical signs of COVID-19 infection were included. The detailed demographic information can be found in Table 1.
Recruitment	Patients admitted to the University Hospital Regensburg between March and July 2020 were enrolled after testing positive for SARS-CoV-2 RNA by qPCR. Additionally, 42 adult healthy volunteers without any clinical signs of COVID-19 infection attending the Universtity Hospital of Regensburg for blood donation were enrolled. Informed consent was obtained by trained staff and sample collection commenced immediately upon study enrollment. There might be a small bias as healthy volunteers were younger than COVID-19 patients.
Ethics oversight	Research Ethics Committee from the University Hospital Regensburg (Study and Approval Number: 20-1785-101)

Note that full information on the approval of the study protocol must also be provided in the manuscript.

Flow Cytometry

Plots

Confirm that:

- [x] The axis labels state the marker and fluorochrome used (e.g. CD4-FITC).
- [x] The axis scales are clearly visible. Include numbers along axes only for bottom left plot of group (a 'group' is an analysis of identical markers).
- [x] All plots are contour plots with outliers or pseudocolor plots.
- [x] A numerical value for number of cells or percentage (with statistics) is provided.

Methodology

Sample preparation	Anti-coagulated fresh blood samples (100 µl) or cells after whole blood stimulations were incubated with various panels of the following directly labeled monoclonal antibodies for 20 minutes at 4°C. Subsequently, samples were treated with FACS Lysing Solution (BD Bioscience) for 10 min, washed with 0,9% NaCl, centrifuged, resuspended in 0,9% NaCl together with FACS counting beads (Invitrogen) and acquired with a BD FACSCanto II flow cytometer. For analysis of cultured PBMCs no erythrocyte lysis was performed.
Instrument	BD FACSCanto II flow cytometer
Software	GraphPad Prism 8 Software
Cell population abundance	Cells populations were reported as a number or as concentration of the patient´s blood sample (cells/µl x 1.000). The MFI of surface markers was shown in absolute or in % of control.
Gating strategy	SSC-A and FSC-A parameters were used to select lymphocytes, monocytes and neutrophils from whole blood. Singlets were seperated based on SSC/FSC parameters. Lymphocytes were gated based on SSC-A and FSC-A to identify pDCs (CD304), basophils (CD11b, CD123), lymphocytes (CD3/CD4/CD8/CD19). Monocytes were gated based on SSC-A and FSC-A to identify CD14+ monocytes (CD14,CD16) and CD16+ monocytes (CD16, CD123). Neutrophils were defined using CD11b and CD16. The full gating path is included in Supplement Figure 10.

- [x] Tick this box to confirm that a figure exemplifying the gating strategy is provided in the Supplementary Information.
